# Examining the effects of passive and active strategies on behavior during hybrid visual memory search: evidence from eye tracking

**DOI:** 10.1186/s41235-019-0191-2

**Published:** 2019-09-23

**Authors:** Jessica Madrid, Michael C. Hout

**Affiliations:** Department of Psychology, New Mexico State University, P.O. Box 30001 / MSC 3452, Las Cruces, New Mexico 88003 USA

**Keywords:** Visual search, Eye movements, Hybrid visual memory search, Search strategies

## Abstract

Hybrid search requires observers to search both through a visual display and through the contents of memory in order to find designated target items. Because professional hybrid searchers such as airport baggage screeners are required to look for many items simultaneously, it is important to explore any potential strategies that may beneficially impact performance during these societally important tasks. The aim of the current study was to investigate the role that cognitive strategies play in facilitating hybrid search. We hypothesized that observers in a hybrid search task would naturally adopt a strategy in which they remained somewhat passive, allowing targets to “pop out.” Alternatively, we considered the possibility that observers could adopt a strategy in which they more actively directed their attention around the visual display. In experiment 1, we compared behavioral responses during uninstructed, passive, and active hybrid search. We found that uninstructed search tended to be more active in nature, but that adopting a passive strategy led to above average performance as indicated by a combined measure of speed and accuracy called a balanced integration score (BIS). We replicated these findings in experiment 2. Additionally, we found that oculomotor behavior in passive hybrid search was characterized by longer saccades, improved attentional guidance, and an improved ability to identify items as targets or distractors (relative to active hybrid search). These results have implications for understanding hybrid visual search and the effect that strategy use has on performance and oculomotor behavior during this common, and at times societally important, task.

## Significance

People routinely perform tasks whereby they must look for many items at once; for instance, scanning the grocery store for all the items on one’s list. Moreover, professional visual searchers such as airport baggage screeners are tasked with the difficult job of looking for many items at once (e.g., weapons and all manner of prohibited items), and must be both fast and accurate at their jobs. In these scenarios, the task requires a visual search through the local environment (e.g., the grocery store, an x-ray of luggage), and a simultaneous search through memory (e.g., the list of items to be purchased, the set of all prohibited items). Our research suggests that during such tasks, it may be in the searcher’s best interest to adopt a passive approach, whereby sought-after items are allowed to “pop into focus,” rather than a more active approach, whereby the searcher tries to effortfully direct their attention around in space. The specific circumstances in which passive strategy adoption is optimal, however, will depend on the extent to which the searcher can tolerate a small decrement in accuracy in exchange for a large increase in speed.

Many situations in daily life call upon us to search through a list of items in memory while also searching for these items in the visual environment. For instance, imagine that you are part of a search and rescue team looking for an individual lost in a heavily wooded area. Anything left behind by this individual could serve as a clue to their whereabouts. In this instance, you must search through your memory of potentially useful (or likely) clues - food wrappers, articles of clothing, vehicle keys, notes, maps, pill bottles, camping gear, etc. - while also scanning for these items in the visual environment. Alternatively, imagine you have the important and high-stakes profession of airport baggage screening. Here, under considerable pressure of time and performance, you must search through complicated scans of travelers’ luggage, simultaneously looking for filled water bottles, guns, knives, improvised explosives, ammunition, and all other manner of dangerous and prohibited items. These types of tasks are referred to as hybrid visual memory search, or more recently (and simply), “hybrid search” (Schneider & Shiffrin, 1977; Wolfe, 2012). While the question of how people search for several objects at the same time has been studied in the past (Schneider & Shiffrin, 1977; Sternberg, 1966), these investigations have typically focused on a relatively small number of target items (i.e., 1–6 items).

Recently, researchers have delved into how people search for as many as 100 distinct targets at once (Wolfe, 2012). However, no one has yet examined how adopting a specific cognitive strategy affects search performance during hybrid visual search. This is an important gap in the literature to fill, as the high demands of a hybrid search task may be lessened - or, the various sub-components of search behavior like attentional guidance or object identification may be improved - through the adoption of a particular type of strategy. It is also especially important to understand the potential impact of strategy use for professional visual search scenarios broadly construed, as adopting simple cognitive strategies may be an efficient and cost-effective way to improve performance during societally important search tasks. Past research indicates that adopting a passive search strategy (i.e., letting a search target “pop” into mind) rather than actively pushing one’s attention around a display, leads to search behavior that effectively balances speed and accuracy (Smilek et al., 2006a; Smilek et al., 2006b; Watson, Brennan, Kingstone, & Enns, 2010), particularly during difficult search tasks. Smilek et al. (2006b) speculated that passive search strategies reduce reliance on cognitive control, and instead induce more rapid and automatic attentional processing during search. Prior research on visual search strategies has often focused on explicit scanning methods (Auffermann, Little, & Tridandapani, 2015; Nickles, Sacrez, & Gramopadhye, 1998; Pradhan, Pollatsek, Knodler, & Fisher, 2009). For example, Auffermann et al. (2015) implemented a chest radiograph search paradigm in which observers were instructed to systematically move their eyes from the center of the image out towards the edge while looking for cancerous nodules. One goal of the current paper is to determine if a strategy that does not specify a particular scanning pattern will also beneficially affect visual search performance.

Visual search has long been a useful way to investigate the distribution of visual attention. For instance, Treisman and Gelade’s (1980) theoretical framework, *Feature Integration Theory* (FIT), investigated how attention is deployed during conjunctive visual search tasks. More recently, a number of researchers have acknowledged the flexible and adaptive nature of attentional deployment (Belopolsky & Theeuwes, 2010; Belopolsky, Zwaan, Theeuwes, & Kramer, 2007; Treisman, 2006). Treisman (2006) proposed that the type of information an observer can gather about stimuli in a visual array depends on the size of the attentional window. For example, focused attention provides information about the features of a particular object, while distributed attention provides more global information about the scene. Germane to the current study, it may be the case that adopting a specific search strategy affects the size of the attentional window, and therefore the way that attention is distributed during hybrid search.

### Passive and active search strategies in visual search

Several previous studies have found that participants who are given an explicit cognitive strategy display marked differences in visual search performance (Smilek et al., 2006b; Watson et al., 2010), object categorization (Jacoby & Brooks, 1984; Whittlesea, Brooks, & Westcott, 1994), and executive function (Bourrier, Berman, & Enns, 2018), compared to uninstructed performance. Of particular interest to the current investigation are those studies that have investigated the distinction between passive and active strategies in visual search. For example, in work conducted by Smilek et al. (2006a), participants were trained to associate verbal labels (e.g., “elephant”, “pencil”) with simple, meaningless shapes. Participants were then asked to identify and localize the unique shape in an array of distractors. This experiment was intended to conceptually replicate the relationship between the categorical membership of a search item and search efficiency, using a novel approach. However, the anticipated effect was uncovered only when Smilek, Dixon, and colleagues performed a series of post-hoc tests on the trials, wherein participants reported spontaneously adopting a “passive” search strategy, in which they let the target item “pop out” by exerting less effort to actively locate it. In a follow-up experiment (Smilek et al., 2006a), participants were asked to complete the same aforementioned task, but were explicitly instructed to adopt either an active or passive search strategy. Results showed that the categorical membership of both targets and distractors only influenced search efficiency (defined in this case as the slope of the (RT) time by set size function) when participants utilized a passive search strategy. In light of these findings, the authors proposed that passive search strategies lead to an increased (and unconscious) tendency towards parallel processing.

Having observed the beneficial effects of a passive search strategy in several studies, Smilek et al. (2006b) designed an experiment to test whether the differences seen in active and passive strategies were dependent on the difficulty of the visual search task. This question closely relates to how strategy instructions influence behavior. If a passive strategy influences the amount of cognitive control an observer exerts, then engaging in a difficult search task should encourage more task control. Therefore, adopting a passive search strategy should reduce this control to a greater extent than an active strategy. In experiment 1, Smilek, Enns, and colleagues (2006b) asked observers to use either a passive or active strategy while searching for a circle that had a gap on one side; distractors were circles that had gaps on both sides. The difficulty of the task was manipulated by varying the width of the gap on the target circle (i.e., smaller gaps are more difficult to detect). In order to assess if a speed-accuracy tradeoff was present, the authors used a combined measure of accuracy and RT called inverse efficiency, which is calculated by simply dividing mean correct RT by the mean proportion correct (see Townsend & Ashby, 1983). The results showed that a passive strategy led to better inverse efficiency scores when the task was comparatively difficult, supporting the hypothesis that strategy influences search by relaxing cognitive control.

In experiment 2, the authors (Smilek et al., 2006b) probed this hypothesis further by actively inhibiting the amount of executive control that was available during the search task. To accomplish this, observers in a dual-task condition were asked to remember an arrangement of circles with gaps before each search trial. After the search task, they were prompted to indicate if a test display matched the arrangement of circles that was viewed at the beginning of the trial. Observers in a single task condition were instructed to simply ignore the memory displays. All observers engaged in both difficult and easy search tasks. Importantly, explicit search strategies were not provided. Instead, the difficulty of retaining the circle array in memory during the dual-task condition was intended to mimic the loss of executive control that is presumably given up during passive search. Analyses revealed that engaging in a simultaneous memory task improved inverse efficiency scores for only the difficult search trials, a result that suggests better inverse efficiency can stem from decreased executive control. Therefore, it may be the case that adopting a passive search strategy creates a similar tendency towards automatic, parallel processing. The notion of parallel processing in visual search is a notoriously difficult issue to disentangle (see Cave & Wolfe, 1990; Godwin, Walenchok, Houpt, Hout, & Goldinger, 2015; Thorton & Gilden, 2007), and it is not the focus of the current work to make a distinctly parallel vs. serial designation regarding search performance. In this context, the term as used by Smilek et al., (2006b) simply refers to a wider processing of target meaning and category membership, and may therefore also be referred to as parallel selection*.*

A more recent study by Watson et al. (2010) analyzed the effect of search strategy on oculomotor behavior. Participants were assigned to either a passive or active-search condition, in which their eye movements were recorded while they searched for a unique target. Stimuli were the same circles with missing gaps that were used in Smilek et al. (2006b), and results showed that search performance produced a similar pattern of behavior: RTs were shorter, but accuracy was lower for the passive-search condition. A speed-accuracy trade off was ruled out, the authors argued, through the calculation of the inverse efficiency score for both groups; the logic behind this measure is to account for speed-accuracy tradeoffs by simultaneously considering speed and accuracy in a single measure (Townsend & Ashby, 1983). The analysis of inverse efficiency determined that passive search was more efficient overall, relative to active search, despite the small decrement in accuracy in the passive searchers.

More importantly, as concerns eye movements, the authors found that passive searchers were more likely to fixate the target item when three or fewer saccades were made, and were less likely to make additional saccades once the target had been fixated upon. Passive search was also linked to longer initial saccade latency, and a faster rate of response once the target had been fixated upon. Regression analysis was conducted in order to determine how strategy instructions affected oculomotor behavior at the individual level. Importantly, a different set of eye-movement behaviors characterized efficient search in the two strategy modes. For both search modes, the time between the first saccade to the target and the response to the target was the best predictor of inverse efficiency. However, better inverse efficiency for passive searchers was characterized by larger saccadic amplitude, whereas better inverse efficiency for active search was associated with a higher rate of saccades. Watson et al. (2010) emphasized that these results were consistent with a “*looking* versus *seeing*” explanation, proposing that passive searchers are better able to process the information gathered from each fixation, whereas active searchers tend to prioritize the scanning of new spatial locations.

While the beneficial effects of passive search appear to be robust, thus far, they have only been tested on a very narrow range of simple visual search tasks. Moreover, they must be interpreted with caution, because they tend to be accompanied by a small decrease in task accuracy. More ecologically valid research that focuses on training observers to adopt a specific search strategy has often concentrated on teaching novices to scan visual arrays using a particular search pattern (or “scan path”; see Kramer, Porfido, & Mitroff, 2019 for a review). However, as pointed out by Kramer et al. (2019), it can be difficult to draw conclusions about the generalizability of search strategy research due to the fact that strategies are often tailored to a very specific type of search task (e.g., radiologists searching x-rays). More general cognitive strategies such as active and passive search have yet to be studied in complex tasks such as hybrid visual memory search, or to have their potential for speed-accuracy tradeoffs more closely scrutinized; we accomplish both of these things in the current study.

### Hybrid search

Recently, researchers have begun to address the complex issue of how visual attention and memory interact by having participants memorize and search for a large number of specific, photorealistic target items. Importantly, these types of hybrid search paradigms have observers look for a number of targets that far exceeds what is widely accepted as the capacity of working memory (Cowan, 2001, also see Miller, 1956). For instance, observers in Wolfe’s (2012) experiment began by memorizing 1, 2, 4, 8, or 16 targets. During each trial, participants searched through a visual array of 1, 2, 4, 8, or 16 random distractor items, and indicated the presence or absence of a target by key press. Analysis revealed that RT increased in a linear manner with visual set size, but increased linearly with the logarithm of memory set size. Using this pattern of results, Wolfe (2012) successfully extrapolated to more items, accurately predicting RTs for tasks that involved looking for a memory set of 100 potential targets.

This logarithmic pattern has also been extended to search for categories of objects rather than search for specific items (e.g., Cunningham & Wolfe, 2014). The use of categorical rather than pictorial targets provides further ecological validity to this experimental paradigm, as real-world search often involves looking for a broadly defined target rather than a specific instance of a target (i.e., any apples at the grocery store as opposed to a specific apple, or any coffee mug in the cabinet; see Hout, Robbins, Godwin, Fitzsimmons, & Scarince, 2017). Additionally, during such “categorical search,” observers cannot simply rely on familiarity with a particular exemplar in order to identify a target. Instead, they must rely on stored mental representations to guide their search. In Cunningham and Wolfe (2014), observers were asked to memorize 1, 2, 4, or 8 categories, which were presented as words. Observers were tasked with locating one target from any of the memorized categories in subsequent search displays. The results showed that while the task was more difficult than searching for specific pictures of objects, RTs still increased linearly with the logarithm of memory set size.

Although hybrid search has been studied in a variety of contexts, using varied stimuli and search conditions, the mechanisms driving this complex form of search remain somewhat unclear. Wolfe, Boettcher, Josephs, Cunningham, and Drew (2015) suggest the following three-step system in order to explain the interaction of memory search and visual search. The authors note that these stages should not be thought of as sequential, but rather as occurring in simultaneous stages. In the first phase, items are selected from the visual display as possible targets. Here, selection is assumed to be serial and guided based on the relevant perceptual features of items in the memory set. Phase two involves the identification and categorization of the selected items. Selected items are filtered through an identification “pipeline” of sorts, to be categorized according to the contents of long-term memory. Importantly, Wolfe et al. (2015) speculated that this is a “massively parallel” process in which many items are selected for search through long-term memory. The useful metaphor of a carwash is used to describe this process (p. 73). Cars may enter a car wash in a serial fashion, but as the cleaning process takes several minutes, multiple cars may be in the wash tunnel at the same time. In the same way, multiple items from a visual array may be in the process of identification at the same time. In the third phase, the outcomes of the identification phase are tested against the items in the memory set to determine a positive or negative match. Wolfe proposes that this search is logarithmic through activated long-term memory (ALTM). Note that while Cowan’s (1988, 1995) original concept of ALTM argues against the existence of a limited short-term memory store, here it is simply used to capture the idea of a separately searchable portion of long-term memory. This memory search leads to either a target-present/target-absent decision, or a return to phase one and additional visual search.

### The current study

Phase two of Wolfe’s proposed system describes a highly parallel information processing stage in which many objects are simultaneously identified and categorized. Recall that in Watson et al. (2010) the authors posited that passive search is characterized by a focus on “seeing” (i.e., processing the information gained from a fixation) rather than “looking” (i.e., prioritizing eye movements to new locations). Therefore, there seems to be an emphasis on highly effective information processing both when people perform hybrid search, and when they adopt passive strategies during search for fewer items.

Furthermore, hybrid search tasks meet some of the same conditions outlined by Smilek et al. (2006b) as particularly important for a passive search strategy to yield beneficial effects. Specifically, hybrid visual memory search is a relatively difficult task. As opposed to searching through simple arrays for a singular target, hybrid search requires an observer to search for a very large number of potential target items in arrays of similar distractors. This task can be made even more difficult by asking observers to search categorically (Cunningham & Wolfe, 2014). Additionally, observers must search through the target items stored in activated long-term memory as well as control their attention and eye movements in order to search effectively through the visual array. These concurrent tasks may be enough to encourage a loosening of executive control, and subsequently, the use of a more passive search strategy (see Smilek et al., 2006b). Based on these arguments, we hypothesized that observers in hybrid search tasks may naturally adopt a passive strategy (i.e., with no specific instructions to do so by an experimenter) in order to effectively cope with the high task demands.

Alternatively, it is possible that observers naturally deploy their attention around the array in a manner more similar to active visual search. This possibility would align more closely with the description in Watson et al. (2010) of an emphasis on “seeing” or prioritizing eye movements to new locations. Wolfe, Drew, and Boettcher (2015) describe the first stage of hybrid search as involving the guided selection of a display based on the basic features of the target items held in memory. Indeed, one of the most interesting ambiguities surrounding an explanation of the mechanisms driving hybrid search is how such a search could be guided in the first place. It is generally acknowledged that search is guided based on the perceptual features of a target item (Egeth, Virzi, & Garbart, 1984; Hout & Goldinger, 2015). In hybrid search, however, observers are required to search for a large number of targets, each with its own set of characteristic features. It therefore seems improbable that hybrid search could be simultaneously guided by the features of every target in memory. However, Cunningham and Wolfe (2014) found evidence that RTs in a hybrid search task increase as a function of how many items in the visual array share features with the target items in memory. Adding items to the display that did not share overlapping perceptual features (i.e., an alphanumeric character distractor when the target was a specific animal) did not incur a cost in RT, suggesting these items were never selected for processing - a compelling argument for the influence of guidance during hybrid search.

There is no definitive link between guided search and active search. However, these ideas share a number of similarities that suggest some degree of similarity between the two. In guided search, attention is directed based on the salient features of items in the visual environment. Active search strategy instructions ask observers to deliberately direct their attention while searching for a target item. Deliberate search could take the form of selecting items at random, or it could be the case that observers rely upon the perceptual features of their targets in order to “guide” their selection process.

The primary aim of the current investigation was to determine if observers in a hybrid search task naturally adopt a more passive or more active strategy. Our ultimate goal was to better understand how attention is deployed during this important and frequently conducted task, with a secondary aim of understanding how cognitive strategies could beneficially impact performance and oculomotor behavior.

## Experiment 1

In experiment 1, our observers memorized a relatively large list of target categories, after which they searched for any instance of an item from any of those categories in displays populated with distracting non-targets (i.e., items from non-overlapping categories). After one block of uninstructed search, observers either continued to search without a specific strategy, or were asked to adopt a passive strategy or an active strategy. Thus, experiment 1 was designed to provide a baseline of natural search behavior (i.e., search without a specified strategy) to which search using passive and active strategies could be compared. We hypothesized that observers in hybrid search tasks would naturally adopt a more passive strategy. We reasoned that the difficulty of the task, as well as the demands of balancing memory search and visual search would lead to the loosening of executive control characteristic of increased parallel processing (Smilek et al., 2006b). To preview our results, despite our initial expectations, the data seemed to support a more active mode of search on the part of our observers.

### Method

#### Participants

One hundred and twenty students from New Mexico State University participated in experiment 1. Students participated for partial fulfillment of a course requirement or on a volunteer basis. All participants signed an informed consent form prior to participation. Participants were required to have normal or corrected-to-normal vision, be fluent readers of English, and self-report normal color vision.

#### Power analysis

The two articles that most strongly motivated the current research were those by Smilek et al. (2006b), and Watson et al. (2010). We attempted to rely on documented effect sizes in these reports in order to estimate the number of participants we would need for data collection. However, some of the necessary information from these studies is not available in the published work. First, Smilek et al. (2006) do not report measures of effect size. It is possible to calculate effect size from mean squared error (MSE), but only when group means are also reported, which was not the case for RT or inverse efficiency in this article (in those cases, plots of the data were provided, but not precise mean values). Therefore, we estimated effect size from this study using the accuracy data.

We calculated effect size for the effect of strategy (active versus passive) on search accuracy from experiment 1 in the study by Smilek et al. (2006b), using their table of means, sample size, and reported MSE. We established Cohen’s *D* of 3.10 (*f* = 1.55). Using this effect size, we conducted an a priori power analysis in G*Power (Faul, Erdfelder, Buchner, & Lang, 2009; Faul, Erdfelder, Lang, & Buchner, 2007), implementing the statistical test for “ANOVA: Fixed effects, special, main effects and interactions.” We indicated desired power of 0.95, with three groups (as we had active, passive, and no-strategy conditions). Based on these inputs, G*Power indicated that the required sample size was a mere 11 people. We also hoped to conduct a power analysis using data from Watson et al. (2010). Unfortunately, the analysis of variance (ANOVA) results from that paper do not include measures of effect size, nor the necessary information (i.e., tables of means, MSE) to calculate it for ourselves.

We adopted a conservative approach in order to deal with this lack of information. The effect size we calculated from Smilek et al. (2006b) was quite large, so we performed a follow-up power analysis using the standard conventions for large effect sizes. In this instance, we indicated an effect size of *f =* 0.40, which is still a large effect (Cohen, 1988), but considerably less so than the one we computed from the paper by Smilek et al. (2006b). This analysis indicated a required total sample size of 100 participants. Thus, even with this comparatively more conservative approach, the sample size of our study (120) exceeded the sample size necessary to achieve adequate power.

#### Design

Three levels of search strategy (no strategy, passive, active) were manipulated between subjects. There were two blocks of visual search trials. In the first block, observers always searched without explicit instructions to adopt any particular search strategy. In the second block, observers were given instructions to adopt a passive strategy, an active strategy, or continued to search with no strategy instructions at all. The presence of the target during search (absent, present) and the number of items in the visual display (16, 24, 32) were additional within-subjects variables, presented in equal proportions. We used target categories (as opposed to specific pictures) in the experiment because previous research indicated that searching for items from a general category tends to be more difficult than searching for specific pictures (Cunningham & Wolfe, 2014, Exp. 2; Schmidt & Zelinsky, 2009; Wolfe, Horowitz, Kenner, Hyle, & Vasan, 2004; Yang & Zelinsky, 2009). Additionally, a memory set size of 24 categories was selected because it is difficult, but not prohibitively so. Maintaining an appropriate level of task difficulty is important because Smilek et al. (2006b) found that the beneficial effect of adopting a passive search strategy was only seen when the search task was relatively difficult.

#### Stimuli

Our search items were images of real-world objects obtained from the “Massive Memory” database (Brady, Konkle, Alvarez, & Oliva, 2008; Konkle, Brady, Alvarez, & Oliva, 2010; cvcl.mit.edu/MM/stimuli.html; see also Hout, Goldinger, & Brady, 2014). These stimuli are full color photographs of real-world objects with no background. The images we used came from 240 different discriminable categories. Target categories were repeated across both experimental search blocks, and were never reused as distractors. The pool of distractor categories used in block 1 was different (but equal in amount) to the pool of distractors used in block 2, and separate distractor categories were used during practice trials as well. Targets were the same 24 categories for the practice and experimental trials and for both blocks of the experiment.

#### Procedure

**Fig. 1:**
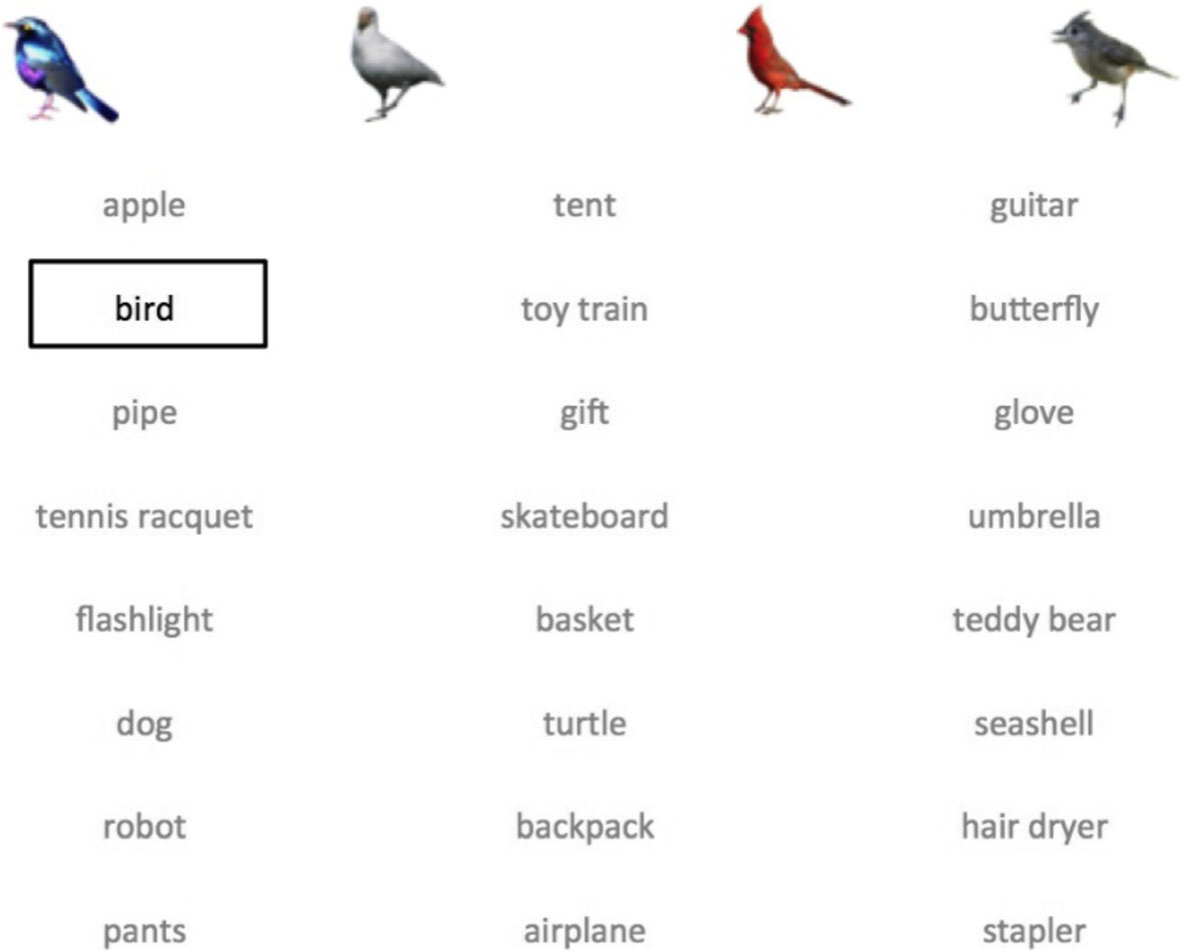
Sample display for the memorization of target categories. Targets were highlighted in black for 3 s each: 4 (out of a possible 16) randomly selected exemplar images corresponding to the highlighted target category appeared at the top of the screen in order to familiarize the participant with the category and eliminate any ambiguity

To begin the experiment, observers were asked to memorize a set of 24 words, which served as the target categories for both experimental visual search blocks. All 24 words were displayed on the screen at the same time in three columns of 8 words each. Each word initially appeared in a gray font. Starting at the top of the left-hand column, each word was darkened to black and surrounded with a box in order to draw the observers’ attention to it for 3 s. As the word was highlighted, four exemplar images appeared at the top of the screen (see Fig. 1). Exemplars were randomly selected from the larger set of 16 items used throughout the experiment. This was done in order to familiarize the observers with examples of what they were required to search for and to ensure that all of the categories were unambiguous. Selection of target categories (from the pool of 240 possibilities) was randomized across participants.

After all of the category words were highlighted, the observer proceeded to a memory test. This consisted of individual words being presented one at a time on the screen. Half of the words were from the target set, and the others were drawn from a larger set of words. Observers were asked to indicate if the word currently displayed was a part of their memory set; accuracy feedback was given after each response. Correct responses were indicated by a green check mark and incorrect responses were indicated by a red “X”. After one iteration of the memory test, the target categories reappeared and the procedure was repeated. If at least 80% accuracy was achieved on two consecutive memory tests, observers moved on to the search trials. If the observer did not achieve at least 80% accuracy on both tests, they were allowed to try both tests one additional time. The threshold of 80% accuracy was obtained from previous research using similar hybrid search paradigms (Wolfe, 2012). No participants required more than two attempts to pass the memory test and overall, mean accuracy for the memory test in experiment 1 was 95%.

**Fig. 2:**
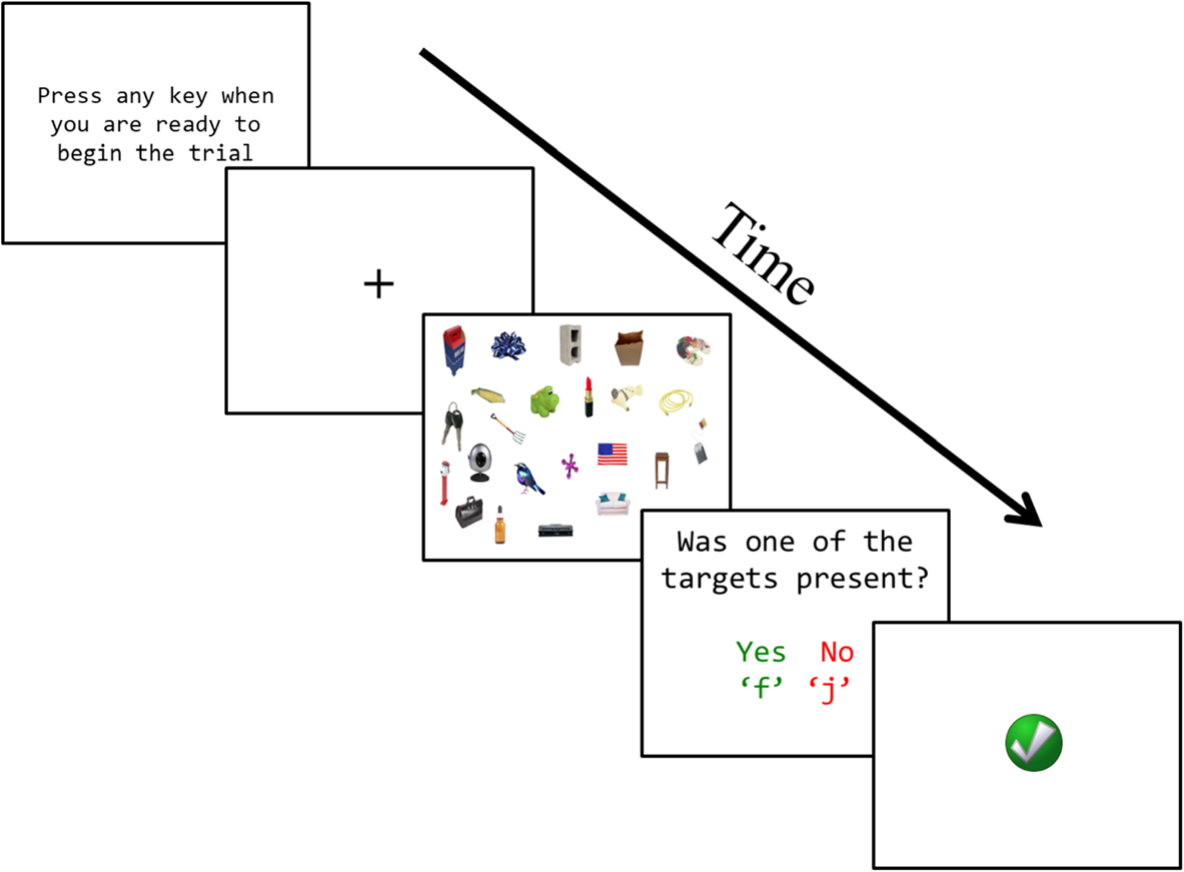
Timeline showing the progression of events in a visual search trial

The first block of search trials established a baseline for search behavior without the influence of a particular search strategy. Observers read a set of instructions that detailed how to perform the hybrid search task, but did not specify a particular search strategy to adopt. During the search trials, a fixation cross was displayed for 500 ms followed by the search display. Observers looked through an array of photorealistic real-world objects for one (and only one) target that represented an item from one of their memorized target categories. Distractors were pulled from the larger set of 240 categories and did not overlap with any of the 24 target categories. The visual set size of the search display varied within subjects between 16, 24, and 32 items; no more than one exemplar from a distractor category was presented on any given trial, thereby creating visual displays with pictures from 16, 24, or 32 different categories. When observers either identified a target item or determined that there was no target present in the display, they pressed the spacebar to end the trial. Participants were not required to localize the target or specify target presence versus absence in order to minimize the amount of motor response selection noise in the RT data (see Hout & Goldinger, 2010, 2012, and 2015 for a similar procedure). A prompt screen then asked observers to indicate (without time pressure) the presence of a target by pressing the letter “f” key, or the absence of all targets by pressing the letter “j” key. Search RT was measured from onset of the display to depression of the spacebar; the response indication screen was not speeded. Feedback was provided after each trial with correct responses indicated by a green check mark and incorrect responses indicated by a red “X” (see Fig. 2). There were 144 trials per block.

The procedure for block 2 was the same as in block 1, save one important detail. Here, observers in the active and passive conditions were prompted to utilize a specified search strategy. Observers in the no-strategy condition simply completed a second block of uninstructed search. Directions detailing the search strategy were presented on the computer screen in front of the participant. The instructions for both groups were adapted slightly from Smilek et al. (2006, pp. 548-549). The passive instructions read as follows (italics are used to emphasize points of comparison between the strategies; text was presented to participants without italics):

“The best strategy for this task, and the one that we want you to use from now on in this study, is to *be as receptive as possible and let the target item “pop” into your mind* as you look at the screen. The idea is to *let the display and your intuition* determine your response. Sometimes people find it difficult or strange to *tune into their “gut feelings”* but we would like you to try your best. Try to respond as quickly and accurately as you can while using this strategy. Remember, it is very critical for this experiment that you *let the target item just “pop” into your mind.”*

The instructions for the active group were as follows:

“The best strategy for this task, and the one that we want you to use from now on in this study, is to *be as active as possible and to “search” for the target item* as you look at the screen. The idea is to *deliberately direct your attention* to determine your response. Sometimes people find it difficult or strange to *“direct their attention”* but we would like you to try your best. Try to respond as quickly and accurately as you can while using this strategy. Remember, it is very critical for this experiment that you *actively search for the target item.”*

These instructions appeared after every ten trials in order to remind the observers to utilize the given strategy.

### Results

A total of eight participants (7%) were removed from the dataset prior to analysis. Five participants were removed for failing to properly follow instructions (i.e., reported using a different strategy than what was assigned or simply pressed keys to advance the screens without searching for the target). Two participants were removed for having mean visual search accuracy greater than 2.5 SDs below the group mean, and one was excluded due to exhibiting mean RT greater than 2.5 SDs above the group mean. Data from a total of 112 participants were included in the analysis.

Recall that block 1 of the search task was always uninstructed in order to establish a baseline for natural search behavior. Observers adopted a passive or active strategy, or continued to search uninstructed in block 2. We therefore analyzed the block 2 data using analysis of covariance (ANCOVA), with participants’ block 1 mean performance used as covariates. This was done to account for pre-existing differences in search performance between observers.

**Fig. 3:**
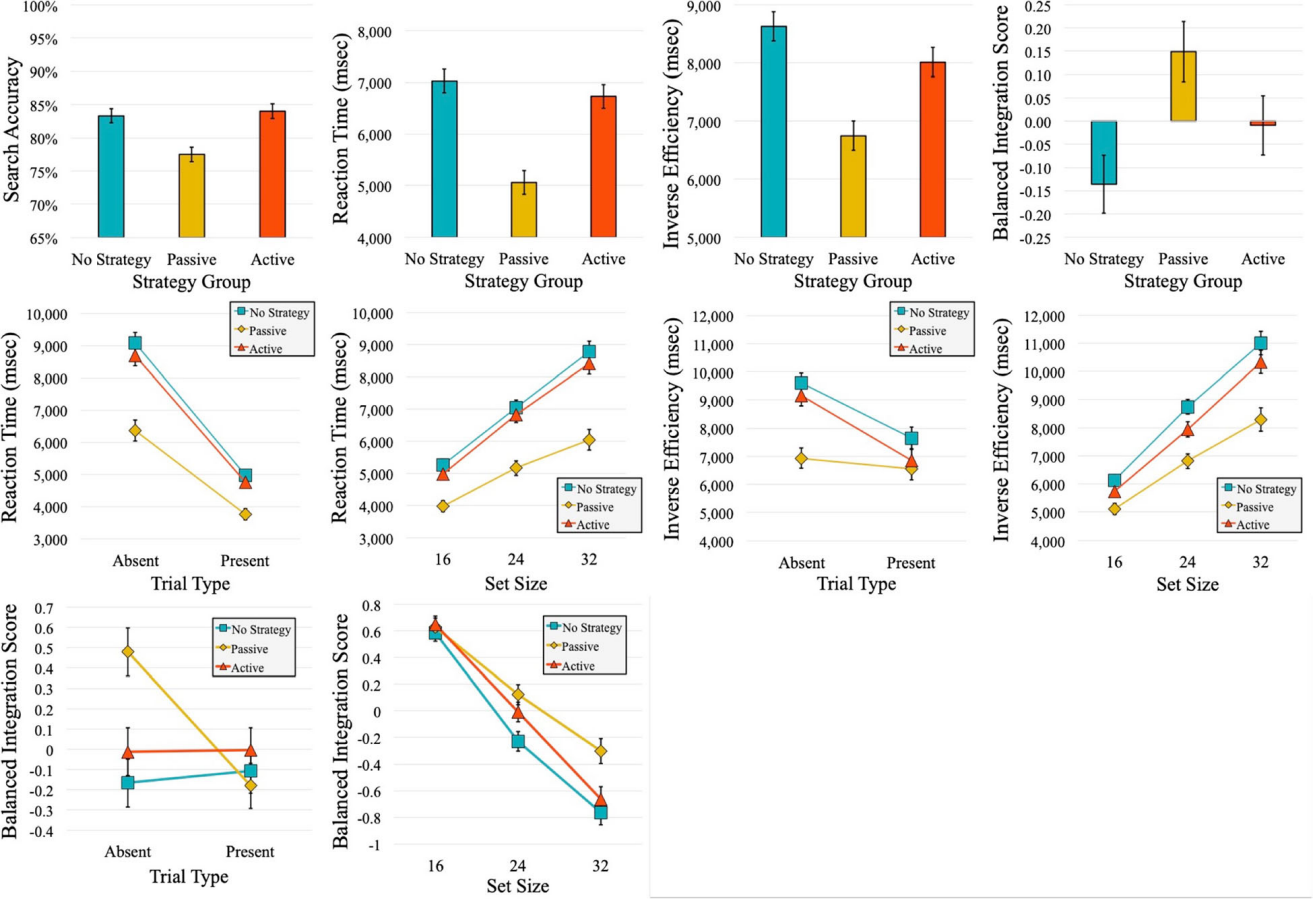
Significant main effects and interactions with strategy, from experiment 1. The top row presents all main effects, and the bottom row presents all two-way interactions. Error bars represent one standard error of the mean (SEM)

Many of the significant main effects found in this study (e.g., trial type, visual set size), as well as many of the interactions, are typical and well-documented in the visual search literature. As such, they are not of central interest here, and in the interest of brevity are not commented upon in any detail. We focused instead on any main effects of (or interactions with) search strategy; such effects are the most important and interesting potential findings, as they most clearly demonstrate the effects of adopting a specific strategy on search behavior. We therefore report only these findings in the body of the text; our entire set of findings, however, can be found in Tables 1, 2, 3 and 4. All significant main effects of and interactions with the strategy factor are plotted in Fig. 3. We used 2 × 3 × 3 ANCOVA to analyze all dependent measures (unless otherwise specified) with trial type (target-present, target-absent) and visual set size (16, 24, 32) as within-subjects factors, and strategy (passive, active, no strategy) as a between-subjects factor. Greenhouse-Geisser correction was used to adjust the degrees of freedom when necessary.

**Table 1:** Full results of analysis of covariance (ANCOVA) for experiment 1

	Accuracy				Reaction time				Inverse efficiency				Balanced integration**			
	*df*	*F*	*η̂* ^*2*^_*<p>*_	*p*	*df*	*F*	*η̂* ^*2*^_*<p>*_	*p*	*df*	*F*	*η̂* ^*2*^_*<p>*_	*p*	*df*	*F*	*η̂* ^*2*^_*<p>*_	*p*
Strategy	2.00	10.61	.16	<.001*	2.00	21.12	.28	<.001*	2.00	14.26	.21	<.001*	2.00	4.95	.08	.009*
Block 1 performance	1.00	133.97	.55	<.001*	1.00	83.55	.44	<.001*	1.00	96.43	.47	<.001*	1.00	108.66	.50	<.001*
Set size	1.98	2.03	.02	.13	1.45	.22	.00	.73	1.47	.15	.00	.79	1.83	332.03	.78	.02*
Set size* strategy	3.96	1.54	.03	.19	2.90	13.01	.19	<.001*	2.94	4.63	.08	.004*	3.67	4.54	.08	.002*
Set size* block 1 Performance	1.98	1.10	.01	.33	1.45	36.23	.25	<.001*	1.47	19.05	.15	<.001*	1.83	5.04	.05	.009*
Trial type	1.00	55.35	.34	<.001*	1.00	4.48	.04	.04*	1.00	.34	.00	.56	1.00	3.30	.03	.07
Trial type* strategy	2.00	1.57	.03	.21	2.00	12.03	.18	<.001*	2.00	3.30	.06	.04*	2.00	4.40	.08	.02*
Trial type* block 1 Performance	1.00	28.45	.21	<.001*	1.00	70.67	.40	<.001*	1.00	.25	.00	.61	1.00	.98	.01	.32
Trial type* set size	1.91	1.78	.02	.17	1.90	3.57	.03	.03*	1.55	1.42	.01	.24	1.96	8.16	.07	<.001*
Trial type* set size* Strategy	3.83	.89	.02	.47	3.80	2.35	.04	.06	3.10	.24	.00	.87	3.91	.31	.01	.87
Trial type * set size *Block 1 performance	1.91	1.97	.02	.14	1.90	19.71	.15	<.001*	1.55	1.02	.01	.35	1.95	.75	.01	.47

*Indicates statistical significance

**Balanced integration score analyzed using analysis of variance

**Table 2:** Full results of analysis of covariance (ANCOVA) for behavioral search performance in experiment 2

	Accuracy				Reaction time				Inverse efficiency				Balanced integration**			
	*df*	*F*	*η̂* ^*2*^_*<p>*_	*p*	*df*	*F*	*η̂* ^*2*^_*<p>*_	*p*	*df*	*F*	*η̂* ^*2*^_*<p>*_	*p*	*df*	*F*	*η̂* ^*2*^_*<p>*_	*p*
Strategy	1.00	5.79	.21	.02*	1.00	32.58	.60	<.001*	1.00	20.66	.48	<.001*	1.00	11.74	.35	.002*
Block 1 performance	1.00	46.52	.68	<.001*	1.00	26.45	.55	<.001*	1.00	26.58	.55	<.001*	1.00	27.60	.56	<.001*
Set size	1.69	3.11	.12	.06	1.37	.16	.01	.77	1.61	2.71	.11	.09	1.86	154.18	.88	<.001*
Set size* strategy	1.69	4.99	.19	.02*	1.37	8.16	.27	.004*	1.61	3.02	.12	.07	1.86	2.73	.11	.08
Set size* block 1 Performance	1.69	1.78	.07	.19	1.37	9.76	.31	.002*	1.61	2.48	.10	.11	1.86	.29	.01	.74
Trial type	1.00	23.59	.52	<.001*	1.00	.43	.02	.52	1.00	.09	.00	.76	1.00	1.74	.07	.20
Trial type* strategy	1.00	1.07	.05	.31	1.00	4.83	.18	.04*	1.00	.47	.02	.50	1.00	2.07	.09	.16
Trial type* block 1 Performance	1.00	14.34	.39	.001*	1.00	8.93	.29	.01*	1.00	.19	.01	.67	1.00	.16	.01	.69
Trial type* set size	1.99	.11	.01	.89	1.64	.54	.02	.55	1.58	4.07	.16	.03*	1.92	.28	.01	.77
Trial type* set size* Strategy	1.99	3.15	.13	.05	1.64	.30	.01	.70	1.58	.32	.01	.68	1.92	2.18	.09	.13
Trial type* set size* block 1 performance	1.99	.32	.01	.73	1.64	4.50	.17	.02*	1.58	4.92	.18	.02*	1.92	5.34	.20	.009*

*Indicates statistical significance

**Balanced integration score analyzed using analysis of variance

**Table 3:** Full results of analysis of covariance (ANCOVA) for oculomotor behavior in experiment 2

	Fixation duration				Saccadic amplitude				Target run index			
	*df*	*F*	*η̂* ^*2*^_*<p>*_	*p*	*df*	*F*	*η̂* ^*2*^_*<p>*_	*p*	*df*	*F*	*η̂* ^*2*^_*<p>*_	*p*
Strategy	1.00	.80	.04	.38	1.00	15.65	.42	<.001*	1.00	18.07	.45	<.001*
Block 1 performance	1.00	12.78	.37	.002*	1.00	35.07	.61	<.001*	1.00	5.11	.19	.03*
Set size	1.90	.91	.04	.41	1.98	1.76	.07	.18	1.62	.17	.01	.80
Set size* strategy	1.90	2.51	.10	.10	1.98	.80	.04	.455	1.62	6.34	.22	.01*
Set size* block 1 performance	1.90	.91	.04	.41	1.98	.12	.01	.888	1.62	1.07	.05	.34
Trial type	1.00	.59	.03	.45	1.00	.78	.03	.39				
Trial type* strategy	1.00	.16	.01	.70	1.00	2.20	.09	.15				
Trial type* block 1 performance	1.00	1.50	.06	.233	1.00	1.21	.05	.28				
Trial type* set size	1.98	1.15	.05	.33	1.49	.50	.02	.56				
Trial type* set size* strategy	1.98	1.77	.07	.18	1.49	1.55	.07	.23				
Trial type* set size* block 1 performance	1.98	1.36	.06	.27	1.49	.65	.03	.48				

*Indicates statistical significance

**Table 4:** Full results of analysis of covariance (ANCOVA) for oculomotor behavior in experiment 2 (cont.)

	Time to first fixation				Target dwell time				Distractor dwell time			
	*df*	*F*	*η̂* ^*2*^_*<p>*_	*p*	*df*	*F*	*η̂* ^*2*^_*<p>*_	*p*	*df*	*F*	*η̂* ^*2*^_*<p>*_	*p*
Strategy	1.00	26.22	.54	<.001*	1.00	12.63	.36	.002*	1.00	7.41	.25	.01*
Block 1 Performance	1.00	14.31	.39	.001*	1.00	7.73	.26	.01*	1.00	9.21	.30	.006*
Set Size	2.00	1.28	.05	.29	1.39	.48	.02	.55	1.93	1.30	.06	.28
Set size* strategy	2.00	5.91	.21	.01*	1.39	1.29	.06	.279	1.93	1.47	.06	.24
Set size* block 1 performance	2.00	.98	.04	.38	1.39	.61	.03	.491	1.93	.90	.04	.41
Trial type									1.00	.07	.00	.80
Trial type* strategy									1.00	4.88	.18	.04*
Trial type* block 1 performance									1.00	1.06	.05	.32
Trial type* set size									1.87	.21	.01	.80
Trial type* set size* strategy									1.87	1.62	.07	.21
Trial type* set size* block 1 performance									1.87	.07	.00	.93

*Indicates statistical significance

#### Accuracy

Here, we found a main effect of strategy (*F* (2, 108) = 10.61, *p* < .001, ${\hat{\eta}}_{<p>}^2$ = 0.16). Accuracy was lowest in the passive-search condition, compared to the active and no-strategy conditions.

#### Reaction time

Only data for correct trials were included in the analysis of RT. Again, we found the crucial main effect of strategy (*F* (2, 108) = 21.12, *p* < .001, ${\hat{\eta}}_{<p>}^2$ = 0.28). Observers in the passive condition exhibited shorter RTs when compared to both the active and no-strategy conditions (both *p*s < .001).

There was a significant interaction between strategy and trial type (*F* (2, 108) = 12.03, *p* < .001, ${\hat{\eta}}_{<p>}^2$ = 0.18)]. We also found an interaction between strategy and visual set size (*F* (2.9, 156.37) = 13.01, *p* < .001, ${\hat{\eta}}_{<p>}^2$ = 0.19). In both interactions, sensible main effects are shown (i.e., longer target-absent than target-present RTs, increasing RT at larger set sizes), and these effects are slightly greater in the no-strategy and active conditions, relative to the passive condition.

#### Inverse efficiency

Past research (Smilek et al., 2006b; Watson et al., 2010) showed that passive search was associated with both shorter RTs and a higher rate of error in comparison to active search. In order to conclude that this was not due to a speed/accuracy tradeoff, an inverse efficiency score (notably distinct from Wolfe’s (1998) definition of “efficiency”) was calculated by dividing the mean RT for correct trials by the mean proportion of accurate responses (see Townsend & Ashby, 1983). This score was calculated for each participant (in each condition) in our experiment, and served to scale RT by accuracy such that inverse efficiency scores during perfectly accurate performance would be equal to the mean RT. Although inverse efficiency does not provide an unbiased measure of overall performance, we chose to include it for several reasons. First, inverse efficiency offers a straightforward interpretation scale; the measure can be thought of as “the average energy consumed by the system,” (Townsend & Ashby, 1983, p. 204) with lower scores indicating more efficient performance. Second, reporting inverse efficiency allows for a more direct comparison between what we have observed and what has previously been documented in this literature. Additionally, we believe that the inclusion of both inverse efficiency and balanced integration scores (see below) provides a transparent picture of our findings and allows us to examine the consistency of our results using several different metrics.

We found a main effect of strategy (*F* (2, 108) = 14.26, *p* < .001, ${\hat{\eta}}_{<p>}^2$ = 0.21), with better inverse efficiency scores in the passive group in comparison to the active and no-strategy conditions (both *p*s < .01). We also found an interaction between strategy and trial type (*F* (2, 108) = 3.30, *p* = .04, ${\hat{\eta}}_{<p>}^2$ = 0.06). Inverse efficiency scores were better for target-present trials in the active and no-strategy conditions, while inverse efficiency was roughly equal for both target-present and target-absent trials in the passive condition. There was a significant interaction between strategy and visual set size (*F* (2.94, 158.73) = 4.63, *p* = .004, ${\hat{\eta}}_{<p>}^2$ = 0.08). Inverse efficiency scores tended to become worse as the number of items in the visual array increased. However, this trend was slightly reduced for the passive condition.

#### Balanced integration score

Inverse efficiency is not without criticism as a method to control for speed-accuracy tradeoffs (Bruyer & Brysbaert, 2011; Liesefeld & Janczyk, 2019). Arguments against its use include the fact that the inverse efficiency measure is not always a good reflection of the relative weights of speed and accuracy (Bruyer & Brysbaert, 2011). More specifically, the relationship between RT and accuracy is not necessarily linear, because the more accuracy scores decrease, the faster the RT scores increase. Recently, Liesefeld and Janczyk (2019) put forth an alternative to inverse efficiency, called the balanced integration score (BIS). The BIS calculation combines speed and accuracy, but in contrast to inverse efficiency, it is designed to give equal weighting to both. Further, this measure has been shown to be less sensitive to speed-accuracy tradeoffs than inverse efficiency and other combined measures of speed and accuracy (e.g., Vandierendonck, 2017, 2018; Woltz & Was, 2006).

Balanced integration scores are calculated by standardizing both the mean RT scores from correct trials and the mean accuracy, and then subtracting standardized RT from standardized accuracy. The means and sample SDs for both RT and accuracy are calculated for each cell, rather than for each condition, to prevent all conditions from having a mean of zero. Balanced integration scores can be interpreted as a measure of how much above or below average the performance was in a given condition or for a given participant when compared to the average of the entire group. For example, positive scores indicate that performance in a given condition were above average overall, with larger scores indicating larger divergence from mean performance (Liesefeld & Janczyk, 2019). After balanced integration scores are calculated they can be subjected to standard statistical tests such as the *t* test and ANOVA. Here, we ran 2 × 3 × 3 repeated measures ANOVA with trial type (target-present, target-absent) and visual set size (16, 24, 32) as within-subjects factors, and strategy (passive, active, no strategy) as a between-subjects factor. Greenhouse-Geisser correction was again used to adjust the degrees of freedom when necessary.

Results showed a main effect of strategy (*F* (2, 108) = 4.95, *p* = .009, ${\hat{\eta}}_{<p>}^2$ = 0.08), with above average performance in the passive-search condition. Additionally, we found a significant interaction between strategy and trial type (*F* (2, 108) = 4.39, *p* = .02, ${\hat{\eta}}_{<p>}^2$ = 0.08). Here, performance was best for the target-absent trials in the passive-search condition. There was also an interaction between strategy and visual set size (*F* (3.67, 108) = 4.54, *p* = .002, ${\hat{\eta}}_{<p>}^2$ = 0.08). Sensibly, performance was best in all three conditions for the lowest visual set size of 16 and declined as the set size grew larger. This trend was diminished for the passive condition, with a smaller reduction in BIS as the set size increased.

### Discussion

The primary goal of experiment 1 was to determine if observers in a hybrid search task tend to naturally adopt a more passive or a more active search strategy. We predicted that if a passive strategy was naturally adopted (i.e., without instruction), then asking observers to use an active strategy would disrupt their performance. Specifically, it would slow performance down, encourage more errors, result in relatively worse inverse efficiency, and lead to below average performance. Additionally, if uninstructed hybrid search is naturally passive, then instructing participants to adopt a passive strategy should not impact their behavior. We speculated that this pattern of results would lend support to the idea that the demands of hybrid search on an individual’s executive control is such that it causes people to search in a more passive manner. Recall that research by Smilek et al. (2006b) suggests that when engaging in visual search, a demanding concurrent memory task leads to passive search and increased parallel processing.

Conversely, we predicted that if observers in hybrid search tasks naturally adopt an active strategy, then asking them to use a passive strategy would lead to search that is faster and less accurate but that is also characterized by above average performance and better inverse efficiency. If this alternative prediction was correct, we reasoned, active search should not differ significantly from the uninstructed search condition. Our results support this alternative hypothesis, indicating a tendency on the part of our participants to naturally utilize an active style during hybrid search. This was evidenced by the high degree of similarity between the active-search condition and the uninstructed search condition in accuracy, RT, and inverse efficiency. However, in comparing performance in the no-strategy condition and the active condition in terms of balanced integration scores, performance in the no-strategy condition was the poorest, while the active condition was closer to the overall group average. Importantly, we found that adopting a passive search strategy led to faster, less accurate search. But we also found performance in the passive-search condition to be above average overall, producing better inverse efficiency and balanced integration scores; evidence that a speed-accuracy tradeoff was not the root cause of the observed trend.

Although experiment 1 did not support our initial idea that hybrid search is naturally passive, our results do provide some evidence that hybrid search does, in fact, place relatively high demand on whichever memory system is responsible for holding the search targets. This idea comes from findings that suggest utilizing a passive strategy only leads to better inverse efficiency when the task is demanding on both memory and visual spatial attention (Smilek et al., 2006b). While it has been speculated by some that hybrid search is largely reliant on familiarity and recognition memory (Guild, Cripps, Anderson, & Al-Aidroos, 2013), the current experiment provides some evidence that the memory component of hybrid visual memory search is relatively taxing. Further evidence of at least some form of recollection memory usage comes from the fact that our observers were given categories (rather than specific pictures) as targets. This argues against the notion that hybrid search is a purely recognition-based task (see also Wolfe et al., 2015).

At this time, our results do not provide evidence that hybrid search involves a “massively” parallel identification and categorization stage of processing. Wolfe et al. (2015) theorized that after a serial, guided selection phase, multiple items pass through an identification “pipeline” and are processed in parallel. Had observers naturally adopted a more passive search strategy, it would have suggested a tendency toward parallel processing. However, it may be the case that the degree of parallel processing in hybrid search exists along a continuum. Perhaps adopting a passive strategy simply increases the degree of parallel processing in this task. Further, parallel processing need not be constrained to the identification phase of hybrid search. Although Wolfe et al. (2015) speculated that visual selection is both serial and guided, passive search could also influence the selection phase as well, allowing a greater number of items to be selected for processing via a broadening of the attentional window (see Olivers & Nieuwenhuis, 2005; Treisman, 2006).

The pattern of results observed in experiment 1 also replicates the finding that passive search is associated with performance that is faster, less accurate, but characterized by better inverse efficiency (Smilek et al., 2006b; Watson et al., 2010) and better-than-average performance (as indicated by balanced integration scores). These findings speak to the reliability of such strategy instructions. Indeed, the considerable shift in search performance in the passive condition suggests a fundamental change in cognitive processing, though the specific processes affected remain unclear. The limited amount of information provided through measures like RT and accuracy does not allow us to dissect what is driving observers’ behavior when using specified search strategies. In experiment 2, we replicated experiment 1, and utilized eye tracking in order to provide richer, converging evidence of processing differences between active and passive hybrid search. Eye-movement data are particularly useful as they allow us to consider evidence other than simply overall speed and accuracy. Because passive search seems to consistently result in shorter RTs and lower accuracy than active or uninstructed search, this raises concerns about speed-accuracy tradeoffs. Although analyses of inverse efficiency and balanced integration scores suggest that a speed-accuracy tradeoff is not driving the behavioral effects of passive search, eye movements may provide additional insight into the potential usefulness of adopting a cognitive search strategy.

## Experiment 2

In order to better understand how strategy adoption affects hybrid search performance, it is necessary to deconstruct measures of performance like accuracy and RT using eye tracking. As previously mentioned, inverse efficiency is an imperfect measurement when there is the possibility of a speed-accuracy tradeoff. We therefore emphasize the importance of eye-movement evidence in experiment 2 to elucidate the effects of cognitive strategies on search performance. We are aware of only one experiment that has looked at the nature of eye movements in hybrid search. Drew and Wolfe (2013) found that distractor dwell time increased in a log-linear manner with memory set size. Additionally, they found that the proportion of items that were fixated upon increased with the number of items in memory.

Watson et al. (2010) identified a relatively clear pattern of eye movements associated with passive and active search. Passive search was characterized by fewer fixations overall, and a tendency to recognize the target more quickly once it had been fixated upon. In contrast, active search was associated with a greater number of rapid saccades to new spatial locations. Watson and colleagues interpreted this to mean that passive search leads to more effective processing of the information available during fixations. It is difficult to predict how these results will transfer from single target search to hybrid search - a much more complex task than that employed by Watson et al. (2010). However, we expected to see a similar trend towards facilitated information processing in the passive-search condition of our study. This would be characterized by fewer processing attempts (i.e., fixations) in order to determine the identity of a target and/or distractor.

Each of the oculomotor measures included in our analysis are defined here: Fixation duration (in milliseconds) was simply the average amount of time the eye remained still (i.e., was not in the process of a saccade); average saccadic amplitude (in degrees of visual angle) was the average length of saccades during search; target run index was the number of items fixated upon prior to fixating upon the target; time to first fixation was the average amount of time until the first fixation was made on the target; and target/distractor dwell time was the total amount of time spent fixating on a target or distractor (regardless of the number of refixations).

Together, these measures provide a thorough picture of how attention is deployed during hybrid search. Fixation duration and saccadic amplitude characterize basic oculomotor behavior, contrasting how long the eyes remain still with how small or how sweeping the movements in between fixations tend to be. Target run index provides insight into attentional selection, quantifying how effectively attention is guided to the target item. Similarly, time to first fixation can be thought of as an indication of how well attention is guided during search. Finally, dwell times for both distractors and targets express how quickly observers are able to verify the identity of a given item.

In experiment 2 we sought to determine if there were meaningful differences in oculomotor behavior between passive and active strategies in hybrid search. We were most interested in determining if eye movements could tell us anything about how attention is deployed when using each strategy, and how this may relate to both target selection and target identification.

### Method

#### Design and stimuli

The design and stimuli of experiment 2 were identical to experiment 1, except for the exclusion of the no-strategy group. After memorizing the target categories, participants completed one block of uninstructed hybrid search followed by one block of search using either a passive strategy or an active strategy.

#### Participants

Twenty-six students from New Mexico State University completed experiment 2. Students participated for partial fulfillment of a course requirement or on a volunteer basis. All participants were required to have normal or corrected-to-normal vision, report normal color vision, and be fluent readers of English. Individuals who participated in experiment 1 were not eligible for experiment 2.

#### Power analysis

In experiment 2, acquiring a sample size as large as that of experiment 1 would have been prohibitively time-consuming (unlike experiment 1, wherein our participants were run through the experiment on “banks” of identical computers, experiment 2 required single participant-sessions on the eye tracker). Thus, in keeping with the majority of eye tracking studies, we anticipated collecting data from a smaller sample. We used the data from experiment 1 to estimate how many participants we would need, and conducted a power analysis in much the same way as we did using estimates from the Smilek et al. (2006b) study. The luxury afforded to us here was that the necessary effect size was readily available to us. We reasoned that the inverse efficiency analysis was the most important finding in experiment 1 because this combined speed and accuracy, and has a documented history of use. As such, we used the effect size of our strategy factor on search efficiency (*η*_*p*_^2^ = 0.209; *f* = 0.514) to conduct an a priori power analysis for experiment 2. We used the same parameter values as we did for the power analysis in experiment 1, save a reduction in the number of groups from three to two (as we no longer had a no-strategy condition). This analysis revealed a required total sample size of 52 participants.

We were unable to acquire such a large sample of participants on the eye tracker, so we conducted a follow-up, post-hoc, power analysis, to determine what level of power we achieved. Here, we used the acquired effect size of strategy on inverse efficiency from experiment 2 (*η*_*p*_^2^ = 0.170; *f* = 0.452), and discovered that we had an achieved power level of 0.60. It may therefore be argued that experiment 2 was, to some degree, underpowered. Nevertheless, even with the smaller sample size required by an eye-tracking study, we still replicated the main effects found in experiment 1.

#### Apparatus

Eye movements were recorded using an Eyelink 1000 eye-tracker (SR Research Ltd., Mississauga, ON, Canada), mounted on the desktop of a 17″ cathode ray tube (CRT) monitor with a refresh rate of 60 Hz. Resolution of the desktop was 1920 × 1200 pixels, and spatial accuracy of the eye tracker was 0.01° of visual angle. Eye movement was defined as a saccade when its distance exceeded 0.5° and its velocity reached 35°/s or acceleration reached 9500°/s^2^. Only the right eye was recorded, but viewing was binocular.

#### Procedure

The procedure was identical to that of experiment 1, with the exception of details pertaining to eye tracking. Participants used a chin rest during all trials and were initially calibrated to ensure accurate tracking. The chin rest was adjusted so each participant’s gaze landed centrally on the computer screen when the participant looked straight ahead. The calibration procedure established a map of the participant’s known gaze position relative to the tracker’s coordinate estimate of that position. The routine proceeded by having participants fixate on a black circle as it moved to nine different positions on the screen. The order of the positions was randomized. Calibration was accepted if the mean error was less than 0.5° of visual angle, with no error exceeding 1.0° of visual angle. Periodic recalibrations ensured accurate recording of gaze position throughout the experiment. Interest areas were defined as the smallest rectangular area that encompassed any given image. The trial procedure was modified to include a gaze-contingent fixation cross. When the fixation cross appeared, participants had to maintain fixation on it for 500 ms, which triggered the search display to appear. In rare circumstances wherein this did not occur within 10 s (because of human error or calibration problems), the trial was discarded and recalibration was performed before the next trial.

### Results

One participant (4%) was excluded from the analysis due to self-reported color blindness. Data from a total of 25 participants were included in the analysis. We used 2 × 3 × 2 ANCOVA to analyze all dependent measures (unless otherwise specified) with trial type (target-present, target-absent), and visual set size (16, 24, 32) as within-subjects factors, and strategy (passive, active) as a between-subjects factor. As in experiment 1, block 2 data were analyzed using mean performance in block 1 (uninstructed search) as covariates to account for pre-existing differences in search performance between observers. Greenhouse-Geisser correction was used to adjust degrees of freedom when necessary. Only data from correct trials were used in the analysis of RT and eye-movement metrics.

**Fig. 4:**
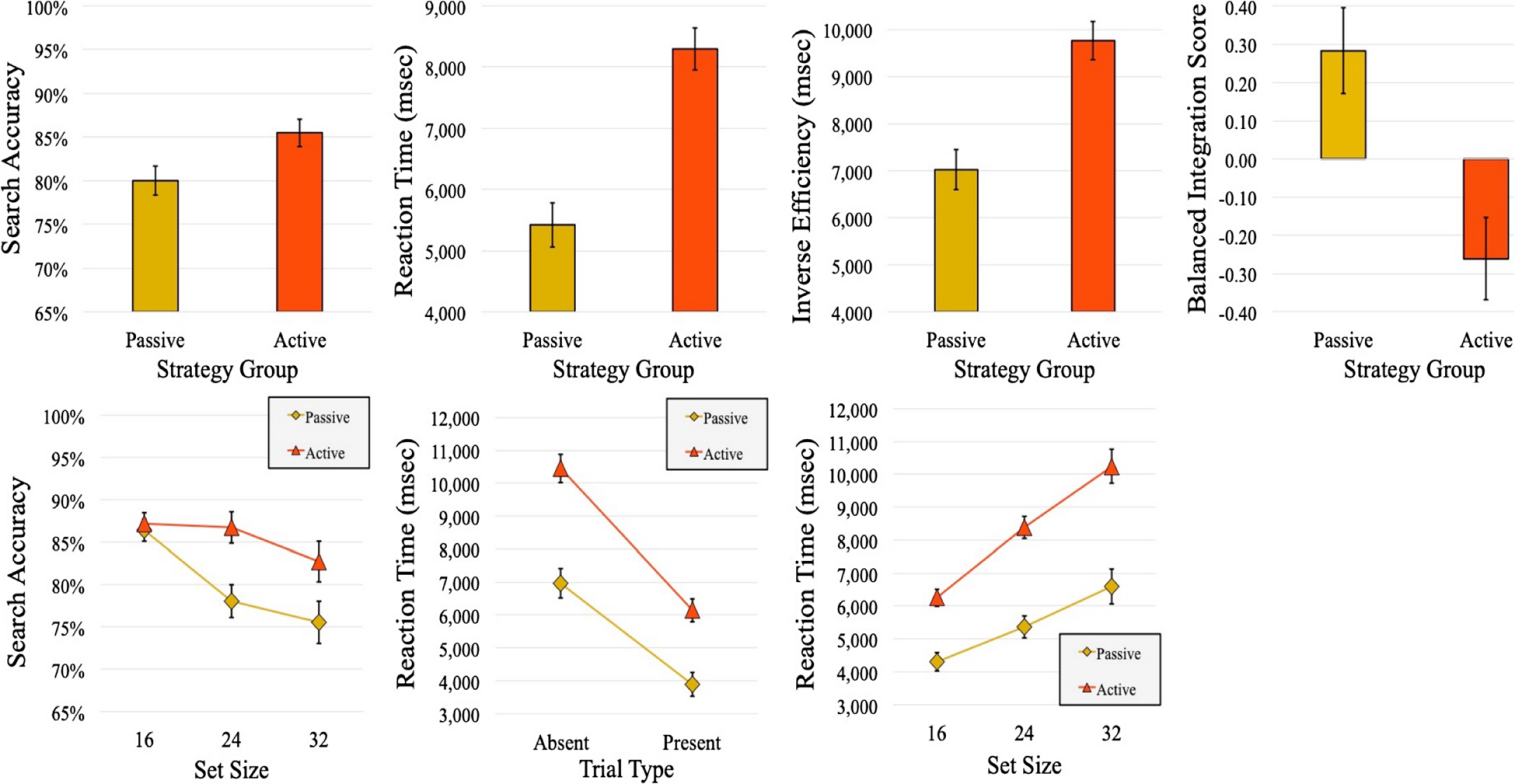
Significant main effects and interactions with strategy, from experiment 2, behavioral measures. The top row presents all main effects, and the bottom row presents all two-way interactions. Error bars represent one standard error of the mean (SEM)

**Fig. 5:**
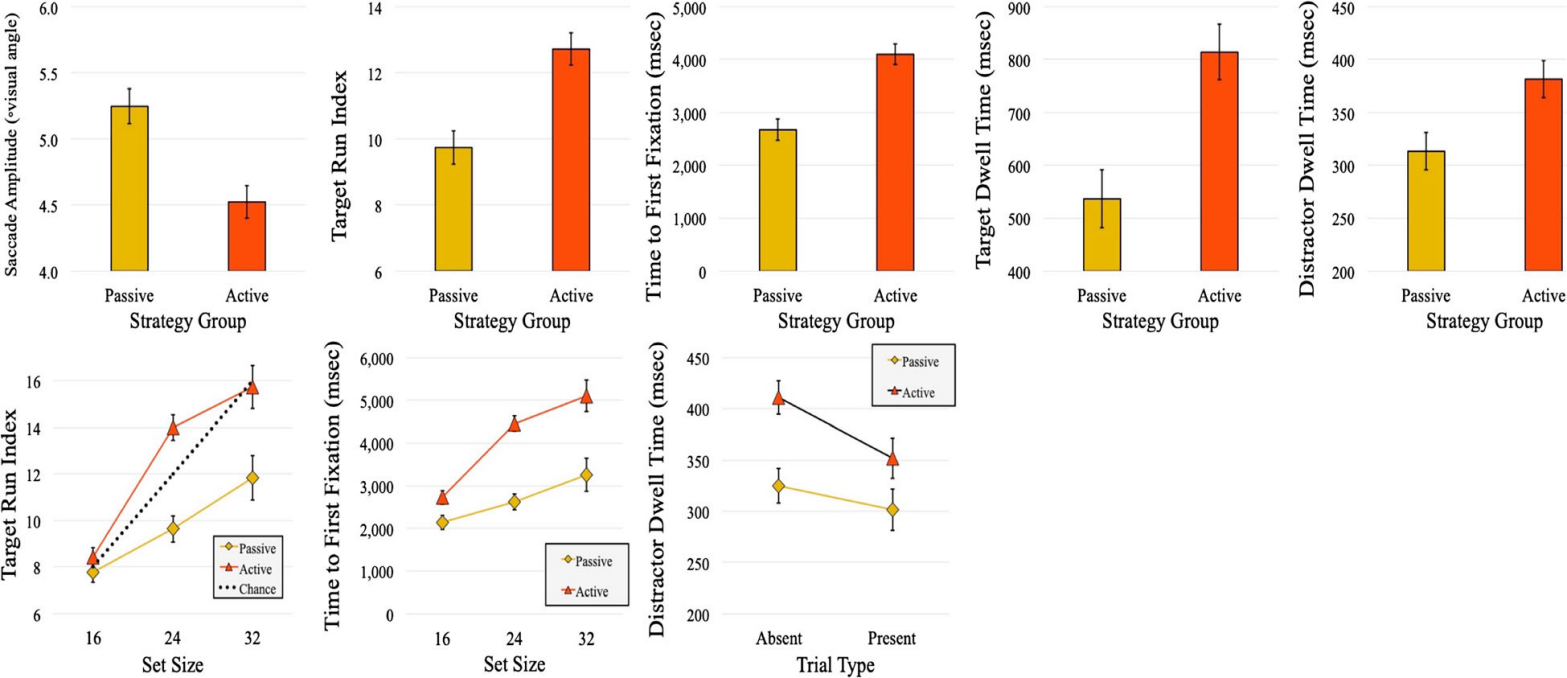
Significant main effects and interactions with strategy, from experiment 2, eye-trackings measures. The top row presents all main effects, and the bottom row presents all two-way interactions. The dotted line in the plot of target run index indicates the number of items that would be expected to be fixated upon due to chance alone, if no attentional guidance was present. Error bars represent one standard error of the mean (SEM)

As in experiment 1, we report only main effects of or interactions with strategy. See Fig. 4 for plots of significant behavioral measures and Fig. 5 for plots of significant eye-tracking measures. The full behavioral results are documented in Table 2 and the full eye-movement results are documented in Tables 3 and 4.

#### Behavioral search performance

##### Accuracy

We found a main effect of strategy on search accuracy (*F* (1, 22) = 5.79, *p* = .03, ${\hat{\eta}}_{<p>}^2$ = 0.21), with lower accuracy in the passive-search condition. There was a significant interaction between strategy and visual set size (*F* (1.7, 37.24) = 4.99, *p* = .02, ${\hat{\eta}}_{<p>}^2$ = 0.19). While accuracy was nearly equivalent for both strategies for the visual set size of 16, accuracy declined more rapidly for the passive condition as the number of items in the array increased.

##### Reaction time

There was a main effect of strategy (*F* (1, 22) = 32.58, *p* = < .001, ${\hat{\eta}}_{<p>}^2$ = 0.60), with shorter RTs in the passive condition. We found an interaction between strategy and trial type (*F* (1, 22) = 4.83, *p* = .04, ${\hat{\eta}}_{<p>}^2$ = 0.18), and a significant interaction between strategy and visual set size (*F* (1.37, 30.18) = 8.16, *p* = .004, ${\hat{\eta}}_{<p>}^2$ = 0.27), in keeping with experiment 1.

##### Inverse efficiency

There was a main effect of strategy on inverse efficiency (*F* (1, 22) = 20.66, *p* = < .001, ${\hat{\eta}}_{<p>}^2$ =0 .48), with more efficient search performance in the passive-search condition than in the active-search condition.

##### Balanced integration score

Recall that the BIS can be interpreted as a measure of how much above or below average performance was in a given condition when compared to the average of the entire group. This means that positive values indicate performance above that of the overall group and negative values indicate performance below the group average. We used 2 × 3 × 2 ANOVA to analyze all dependent measures with trial type (target-present, target-absent) and visual set size (16, 24, 32) as within-subjects factors, and strategy (passive, active) as a between-subjects factor. We found a main effect of strategy (*F* (1, 22) = 11.74, *p* = .002, ${\hat{\eta}}_{<p>}^2$ = 0.35) with above average performance in the passive condition and below average performance in the active condition.

#### Oculomotor behavior

##### Fixation duration

There was no significant main effect of strategy (*F* (1, 22) = 0.80, *p* = .38, ${\hat{\eta}}_{<p>}^2$ = 0.04) on average fixation duration. There were no significant interactions with strategy.

##### Saccadic amplitude

We found a main effect of strategy on average saccadic amplitude (*F* (1, 22) = 15.65, *p =* .001, ${\hat{\eta}}_{<p>}^2$ = 0.42). The average saccadic amplitude was larger in the passive-search condition. There were no other interactions with strategy.

##### Target run index

Trial type was not included as a factor here (or in the time-to-first-fixation or target-dwell-time metrics), as this measure necessitates that we only assess trials with a target present. We found a main effect of strategy on the number of items fixated upon prior to fixating on the target (*F* (1, 22) = 18.07, *p* < .001, ${\hat{\eta}}_{<p>}^2$ = 0.45). Compared to when an active strategy was used, observers in the passive condition fixated on an average of three fewer items before fixating on the target. There was also a significant interaction between strategy and visual set size (*F* (1.63, 35.76) = 9.54, *p* = .007, ${\hat{\eta}}_{<p>}^2$ = 0.22). While target run index was similar for both the passive and active conditions when the visual set size was 16, there was a marked increase in target run index in the active condition for the visual set sizes of 24 and 32. Target run index also increased for the visual set sizes of 24 and 32 in the passive condition, but the increase was more modest.

##### Time to first fixation

There was a main effect of strategy on time to first fixation (*F* (1, 22) = 26.22, *p* = < .001, ${\hat{\eta}}_{<p>}^2$ = 0.54), with observers in the passive strategy condition taking less time on average to make their first fixation on the target. We also found a significant interaction between strategy and visual set size (*F* (1.54, 33.96) = 5.91, *p* = .01, ${\hat{\eta}}_{<p>}^2$ = 0.21). While the average time to first fixation increased with the number of items in the visual array for both conditions, time increased more conspicuously for the set sizes of 24 and 32 in the active condition. The time to first fixation increased in a more moderate fashion in the passive-search condition.

##### Target dwell time

Only target-present trials were included in this analysis. We found a main effect of strategy on target dwell time (*F* (1, 22) = 12.63, *p* = .002, ${\hat{\eta}}_{<p>}^2$ = 0.37), with shorter dwell times on targets in the passive condition. There were no significant interactions with strategy for target dwell time.

##### Distractor dwell time

There was a significant main effect of strategy on average distractor dwell time (*F* (1, 22) = 7.42, *p* = .01, ${\hat{\eta}}_{<p>}^2$ = 0.25). Dwell times were shorter in the passive condition compared to the active condition. We also found an interaction between strategy and trial type (*F* (1, 22) = 4.88, *p* = .04, ${\hat{\eta}}_{<p>}^2$ = 0.18), with a larger increase in dwell time on target-absent trials for active searchers, relative to passive searchers.

#### Analysis of target misses

##### Viewing failures versus recognition failures

Here, we considered that there are two possible reasons why an observer might miss a target. First, they may miss it because they simply failed to direct their attention to it; we refer to this as a viewing failure. Alternatively, they may miss a target because they look at it and yet fail to perceive that it is what they are searching for; we refer to this as a recognition failure (see Hout, Walenchok, Goldinger, & Wolfe, 2015). In order to determine if the passive and active conditions differed from each other in regards to the rate and type of misses that occurred, we analyzed target misses in block 2 using the nonparametric chi-square test of independence. Frequency of errors was used to compute the chi-square test but this was converted to proportions for ease of interpretation (see below).

In the passive condition, viewing failures and recognition failures occurred at almost precisely equal rates (51% viewing failures, 49% recognition failures). However, this distribution was much less even in the active condition, with 23% viewing failures and 77% recognition failures. Therefore, observers in the active-search condition were significantly more likely to commit recognition failures (relative to viewing failures) compared to those in the passive condition (*χ*^2^ (1, *N* = 25) = 47.04, *p* < .001).

##### Recognition failure dwell time

**Fig. 6:**
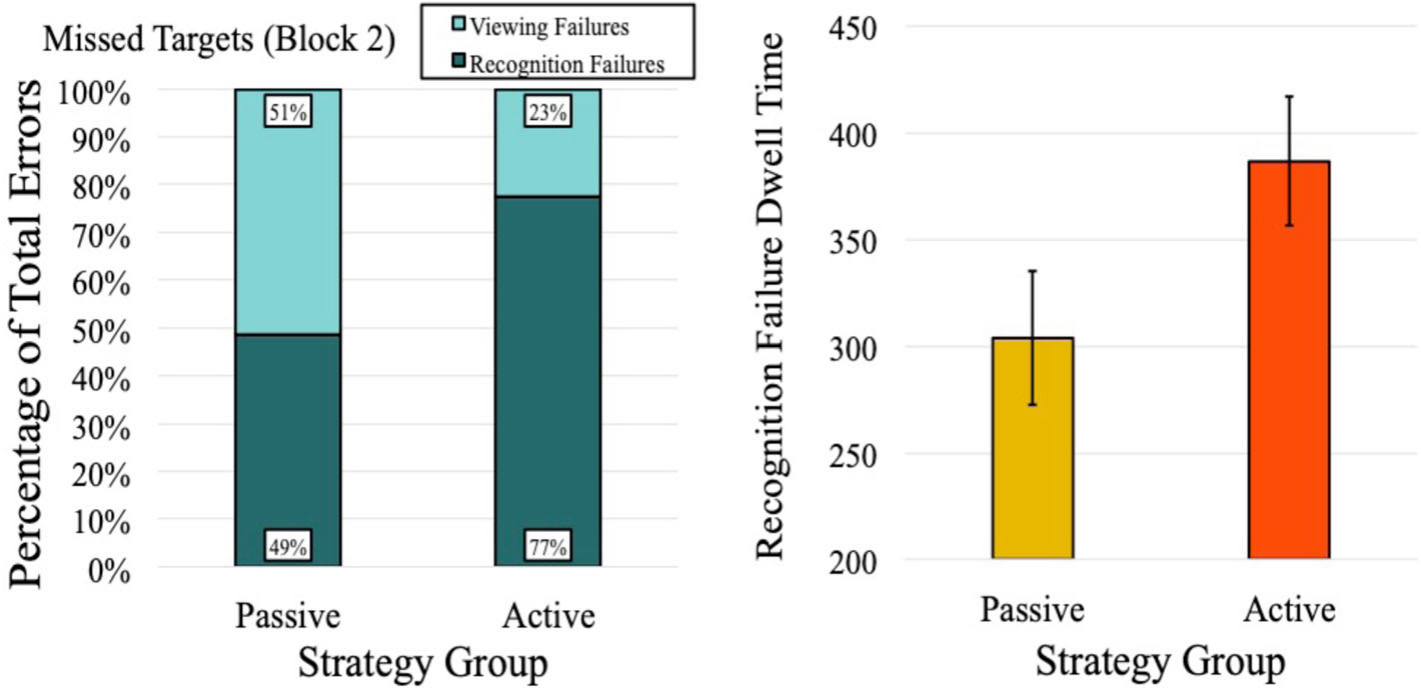
Examination of target misses from experiment 2, derived via eye tracking. The left panel shows the distribution of viewing to recognition failures (across strategy groups). Note that the figure shows proportion of error types, not overall error rates (which varied across groups and were higher for passive searchers). The right panel shows target dwell times when recognition failures occurred. Error bars represent one standard error of the mean (SEM)

Although we found that recognition failures were more likely to occur during active search, it is possible that when such recognition failures occurred, they were due simply to reduced dwell times on the target. That is, perhaps active searchers commit more recognition failures because when they do look at the target, they fail to look at it for long enough to recognize it. To test this possibility, we compared the recognition failure dwell times in the passive and active conditions. Here we found a marginally significant difference (*F* (1, 22) = 3.61, *p* = .07, ${\hat{\eta}}_{<p>}^2$ = 0.14), suggesting that (contrary to our prior suggestion) when recognition failures occurred, observers in the active condition spent longer looking at the targets than observers in the passive condition (see Fig. 6).

### Discussion

The purpose of experiment 2 was to examine how eye movements could inform our understanding of attentional deployment and information processing in passive and active hybrid search beyond behavioral measures like RT, accuracy, inverse efficiency, and the BIS. In experiment 1, we showed that while hybrid search may be naturally active, utilizing a passive strategy leads to noticeable changes in search behavior. In experiment 2, we built on this information by assessing how these behavioral changes are related to the processing of items within a visual array. We hypothesized that passive search would be characterized by eye movements indicative of parallel processing and facilitated decision-making. Our data supported this prediction.

Importantly, we were able to replicate the main effects of strategy on accuracy, RT, inverse efficiency and BIS from experiment 1 despite a reduction in sample size. We found that searchers in the passive condition were less accurate, but faster, and more efficient than active searchers. Additionally, performance in the passive-search condition was above average as indexed by the analysis of the BIS. These results lend further support to the idea that hybrid search may be naturally more active in nature, but also that adopting a passive strategy decreases RTs with only a minimal loss in accuracy. We did not replicate the inverse efficiency and BIS interactions between strategy and visual set size or between strategy and trial type that we observed in experiment 1. We expect that this was the result of reduced statistical power, and acknowledge the need for further replications with a larger sample size. More interestingly, the majority of our eye-movement measures indicated a clear difference between the passive-search condition and the active-search condition.

Target run index analyses, used here as a measure of attentional selection, suggested that observers in the passive condition experienced improved attentional guidance, as they looked at fewer items before finding the target, relative to observers in the active condition. It should also be noted that the number of items fixated upon prior to finding the target can informatively be compared to chance performance; that is, if no attentional guidance is present during search, and selection of items is instead random, we would expect half of the items in the display to be fixated upon prior to the target. In the active-search condition (shown in the bottom-left panel of Fig. 5), it can be seen that active search leads to performance that is no better than would be expected by chance alone, questioning the extent to which active hybrid search is in fact “guided.” By contrast, passive searchers clearly performed better than chance with higher set sizes, suggesting a beneficial impact of passive strategies on attentional guidance.

In regards to decision making, observers in the passive condition were able to more easily verify the identity of items in the visual array as evidenced by the shorter dwell times for both targets and distractors. Watson et al. (2010) posits that passive searchers are able to represent the information obtained from each fixation more “richly” than those using an active strategy (p. 543). However, the mechanism that may be driving this process is unclear. Additionally, it seems unlikely that the more complex stimuli used in hybrid search could somehow be represented more richly when it has been shown that passive hybrid search is both faster and less accurate than natural hybrid search.

Bar (2003) has proposed a cortical mechanism for top-down facilitation of visual object recognition that may be more representative of what is occurring in passive hybrid search. He posits that early visual areas project a partial representation of an input image to the prefrontal cortex. This partial image provides a “gist” of the item and activates expectations about the identity of an input image. These expectations are then back-projected as “initial guesses” to the temporal cortex where they are used in tandem with bottom-up information to identify the object. Facilitation is achieved by limiting the number of interpretations that can be made about an item based on the initial, coarse, visual information. Bar (2003) states, “this rapid process significantly reduces the amount of time and computation required for object recognition” (p. 601). The longer, sweeping, eye movements characteristic of passive hybrid search seem likely to provide the kind of coarse visual information described by Bar (2003) as being involved in this mechanism. Furthermore, the reduced target and distractor dwell times in passive hybrid search also fit well with Bar’s description of reduced computational expenditure.

## General discussion

This investigation was designed to determine if giving people specific cognitive strategies would change their behavior during hybrid search, and if so, to reveal what these changes could tell us about attention and cognitive processing. An additional goal was to test if a generalized strategy that requires little training or attention to specific scan patterns would affect hybrid search performance. We began with the hypothesis that the demands placed on executive control by concurrent memory and visual search caused observers in this task to naturally adopt a more passive search strategy. The loosening of executive control in demanding search tasks has been shown to promote efficient search performance through increasing parallel processing (Smilek et al., 2006b). We reasoned that the remarkably good performance by those in hybrid search tasks (Wolfe, 2012) could be indicative of a natural tendency towards passive search and parallel processing.

In experiment 1, we found that hybrid search seems in fact to be naturally active. Search with no instructions and search in which observers were instructed to “actively direct their attention” to determine their response were extremely similar in accuracy, RT, and inverse efficiency. In terms of BIS, however, no-strategy search and active search were less similar. More interestingly, we found that adopting a passive strategy and “letting targets ‘pop’” into mind led to behavioral deviations from what is done naturally (i.e., without instructions to search in one way or another). Passive search in our study was characterized by faster, less accurate, but ultimately above average performance overall.

We largely replicated our findings in experiment 2. There, we found that adopting a passive strategy led to faster, less accurate, above average search on the part of our participants. More importantly, we found a distinctive difference in oculomotor behavior between passive and active searchers. Passive search was characterized by larger, sweeping saccades, a smaller number of items fixated upon prior to viewing the target, and shorter times to first fixation. Additionally, observers in the passive condition needed less time to identify both distractors and targets.

At present, we cannot definitively explain why passive search causes a change in inverse efficiency and overall performance. It is feasible that processing in passive hybrid search is facilitated through a widening of attentional focus, allowing more items to be selected for further consideration. There has been much research on the influence of arousal, specifically positive and negative affect on the breadth of attentional focus (see Isen, 2000 for a review). Derryberry and Tucker (1994) propose that positive mood is associated with a broader scope of attention. More generally, Olivers and Nieuwenhuis (2005) found that when observers engaged in a distracting secondary activity (i.e., listening to music or doing free-association) they were better able to identify a pair of targets in a stream of rapidly presented images; the classic *attentional blink* paradigm. Observers in this task typically cannot detect the presence of a second target if it follows the initial target closely in time, due to limited attentional capacity (Broadbent & Broadbent, 1987). These results therefore point to a broadened attentional focus. They propose that the concurrent activities induced a “distributed state of mind” (p. 265). It may be the case that adopting a passive search strategy acts in a similar fashion, increasing reliance on automatic processes through a release of executive control.

In addition to a wider focus of attention, it is also conceivable that passive search facilitates the identification of items in hybrid search. We propose Bar’s (2003) theory as a plausible mechanism for facilitated object identification. This theory posits that a top-down mechanism triggered by images with low spatial frequency can aid object recognition by providing a gist or best guess about the identity of an object, thus limiting the possibilities to be considered. Spatial frequency can be thought of as the amount of detail present in an image per degree of visual angle (Bar, 2004). Therefore, an image with low spatial frequency will have less defined, blurrier edges and fewer small details, providing “global information such as orientation and proportions” (Bar, 2003, p. 601). A study by Bar et al. (2006) provides evidence for the utility of this mechanism. Utilizing magnetoencephalography and functional magnetic resonance imaging, Bar et al. (2006) found that object recognition for images with low spatial frequency caused activity in the left orbitofrontal cortex 50 ms earlier than it did in areas of the temporal cortex linked to object recognition. Bar suggests that the orbitofrontal cortex may be involved in the rapid formation of gist for items with coarse visual information. It is possible that the longer, sweeping, eye movements associated with passive search in the current study led to an initial crude encoding, facilitating better recognition when an item was finally fixated upon directly.

## Conclusion

One goal of this research was to determine if cognitive strategies that do not specify a particular scan pattern can affect hybrid search performance. As domain-general strategies, passive and active search have the potential for application across a wide range of professional search scenarios. While it is important to recognize that passive search seems to be accompanied by a modest decrease in accuracy, evidence from eye-tracking metrics suggests that this strategy does nevertheless have the potential to affect both attentional guidance and object recognition in a beneficial manner. In addition to being easy, fast, and affordable to implement, the use of all-purpose search strategies may generalize to novel situations more easily than those previously studied. In contrast to other methods of improving visual search, such as through object identification training or by training searchers to utilize technology (Kramer et al., 2019), simple passive search strategies may be less cognitively demanding, allowing for attentional resources to be better allocated to the search task itself. Although the current state of research on passive search does not allow us to make strong claims about its benefits in real-world search, we believe general cognitive strategies hold great potential for application.

Because the two experiments discussed here were the first of their kind, it will be necessary to conduct more research to further investigate the attentional mechanisms involved in hybrid search, in particular, the causes of above average performance and improved inverse efficiency when utilizing a passive-search strategy. With a better understanding of the mechanisms underlying passive search, it may be possible to develop a strategy that is more highly optimized for high-stakes search. Furthermore, attention and caution should be paid to the potential detriments of passive-search strategies. In real life, high-stakes search scenarios like Transportation Security Administration (TSA) baggage screening prioritize accuracy over speed of search. Clearly, search that is faster but less accurate than natural search may not be a universally useful tool in these circumstances. Depending on the specific scenario, the benefit of enhanced search speed using a passive strategy may be outweighed by the cost of lower accuracy, depending on the extent to which the searcher values finding targets quickly and the extent to which they value perfect (or near-perfect) hit rates. To be clear, the accuracy detriments in our studies were quite small (~ 5% decrease relative to active and uninstructed search), and overall, performance for passive searchers was still quite high (i.e., always above 75%, which is quite good, considering the number of distinct categories that searchers were looking for). Nevertheless, even a small detriment in search accuracy may outweigh the benefits of speed and inverse efficiency in the context of professional search, whereby the cost of missing a single target (i.e., failing to notice a weapon in a traveler’s bag) can be so high. In these cases, a more systematic search strategy, in which observers are asked to use a specific scan pattern (e.g., Auffermann et al., 2015), may be more appropriate.

In other professional search scenarios, however, it should be considered that the small decrement in accuracy may be an acceptable cost when considering the potential benefits of passive search speed, particularly when more than one target may be present in the search environment. For instance, in scenarios wherein multiple targets are present, the identification of a single target may trigger follow-up searches that might otherwise not have occurred. A baggage screener searching luggage with a concealed water bottle and a knife does not have to notice both targets during the initial search; identifying one or the other will trigger the bag to be pulled from the scanner and then manually inspected, likely resulting in the location of both prohibited items. Similarly, a radiologist who notices one abnormality on a chest x-ray may then perform subsequent, slower searches (or consult a colleague for a second view) to make sure that all abnormalities have been identified in the patient.

Moreover, in some scenarios such as search and rescue (SAR) “clue search,” multiple targets may be present, and the benefit of finding any clue quickly could far outweigh the cost of missing one. For example, a SAR “ground pounder” who scours the last known location of a missing hiker for clues to their whereabouts might, given unrestricted time to search, locate a discarded food wrapper, or a hat that was shed by the missing person. In such cases, finding one of these clues quickly may allow the SAR team to make better-informed decisions about the search area, and identify areas of wilderness where the hiker is or is not likely to have travelled. Thus, in this scenario, finding one of these clues quickly may be more beneficial than finding all of them slowly. Put simply, there may be a benefit, in certain professional search scenarios, of finding one of multiple possible targets quickly, even if less than 100% of the targets are identified in an initial search. Future research will be needed to determine if the behavioral effects of passive search extend to multiple target identification, and in general, consideration will need to be given in weighing the relative pros and cons of such search strategies, as a “one size fits all approach” is certainly unlikely to prove fruitful.

Outside of professional search, numerous everyday tasks also involve some form of hybrid visual memory search. Looking for many items in the grocery store or for a large number of friends in a crowd relies on both a search through memory and the visual environment. While people tend to be quite competent at these tasks, there is always room for improvement. Our findings indicate that adopting a passive approach to search may be beneficial when a balance of speed and accuracy is needed. It is still somewhat unclear exactly how passive search leads to more efficient search performance, but our results point to broadened attentional focus and facilitated object identification, and future studies will be directed at better understanding the benefits and pervasiveness of passive and active strategies across a wide range of search scenarios.

## Data Availability

Data cannot be made publicly available because this was not requested at the time of IRB approval. All stimuli and experimental programming can be obtained by emailing the second author (mhout@nmsu.edu).
